# Cefixime induced Steven Johnson syndrome: A case report from Bangladesh

**DOI:** 10.1016/j.amsu.2022.104089

**Published:** 2022-06-28

**Authors:** Abhigan Babu Shrestha, Sajina Shrestha, Prashant Kumar Yadav, Lukash Adhikari, Anuj Yadav

**Affiliations:** a M Abdur Rahim Medical College, Dinajpur, Bangladesh; b KIST Medical College, Imadol, Patan, Nepal; c Patan Academy of Health Science, Lagankhel, Lalitpur, Patan, Nepal

**Keywords:** Adverse drug reaction, Case report, Cefixime, Cephalosporin, Stevens-Johnson syndrome

## Abstract

**Introduction:**

Stevens Jonson syndrome, a type IV mediated hypersensitivity reaction is a rare mucocutaneous disorder accounting for <10% of TBSA. It affects skin, oral mucosa, eyes, esophagus, mouth, pharynx, larynx, skin and genitals. SJS is caused mainly due to drugs, infectious agents, immunization, and radiation therapy.

**Presentation of case:**

We present a case of a 40 years old male who developed SJS after being administered cefixime for a short period. Given the patient's past profile, he was admitted due to RTA and was under treatment with cefixime. Irrespective of any symptoms of SJS in the past, he started developing symptoms soon after being treated with cefixime giving us a clue about cefixime-induced SJS.

**Discussion:**

Steven-Johnson syndrome (SJS) and toxic epidermal necrolysis (TEN) are opposite ends of a spectrum of diseases arising usually from an adverse reaction to medications. The most common drug reactions include penicillin in antibiotics, carbamazepine in antiepileptics and allopurinol in gout treatment in the Asian community. In our case, the patient was under Cefixime for 6 days after which cutaneous manifestations were seen. SJS is a fatal condition, with a global mortality rate stretching between 10% and 34%. The first step in its management is to identify the culprit drug and stop its use. Other is symptomatic, with special attention to airway and hemodynamic stability, wound care, and pain alleviation measures. Medical therapy include corticosteroids, cyclosporine, intravenous immunoglobulin (IVIG), and TNF- α inhibitors.

**Conclusion:**

Cephalosporin group, like cefixime, is a commonly prescribed drug in developing countries due to its efficacy and cost-effectiveness. Therefore, physicians must beforehand be mindful of the consequences of its use and advice patients to visit the hospital with even the slightest cutaneous manifestation.

## Highlights

• Steven Johnson syndrome is a type 4 hypersensitivity reaction to drugs (in our case; cefixime), infectious agents and even vaccines.
• Modified SCORTEN score comprises two more additional parameters to determine the prognosis of this fatal disease.
• Medical therapy include corticosteroids, cyclosporine, IVIG, TNF- α inhibitors. More evidences are required for definite and alternative management of SJS/TEN.

## 1 Introduction

**Fig. 1:**
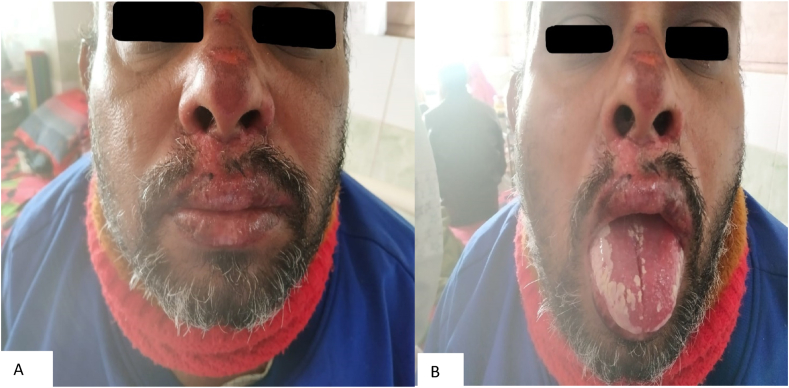
Hyperpigmented reddish-purple macules and areas of desquamation over the face (A) Hemorrhagic crusty erosion of the mucosa of lips and tip of the nose and also whitish lesion over the tongue. . (For interpretation of the references to colour in this figure legend, the reader is referred to the Web version of this article.)

**Fig. 2:**
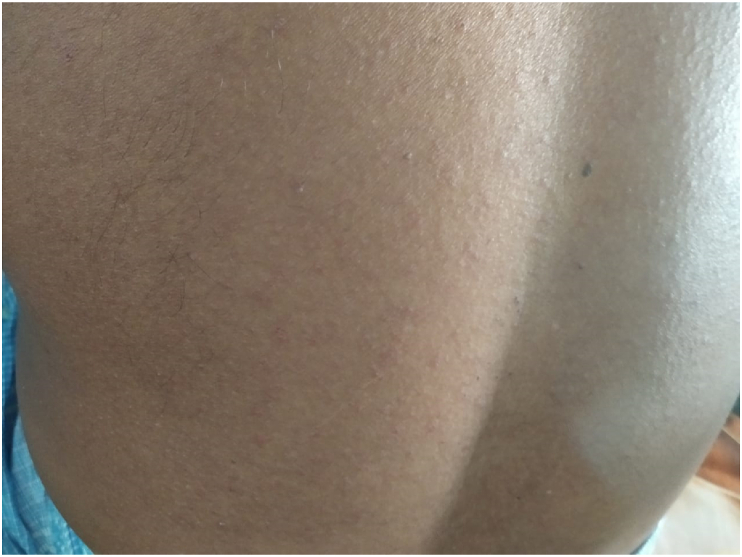
Purpuric macules and papules on the posterior aspect of trunk.

Steven Jonson syndrome (SJS) is a rare but severe form of mucocutaneous disorder characterized by epidermal detachment due to keratinocyte death [1]. It is a delayed hypersensitivity reaction activated by cytotoxic T lymphocytes and natural killer cells releasing various cytotoxic mediators. It accounts for less than 10% of total body surface area affecting the skin, oral mucosa, eyes, esophagus, mouth, pharynx, larynx, skin and genitals [1]. The incidence of SJS and Toxic Epidermal Necrolysis (TEN) is 1.0–6.0 per million and 0.4 to 1.2 million respectively, nevertheless, this is twofold for the Asian people [2]. Causes include drugs, infectious agents, immunization, radiation therapy, graft versus host disease and environmental chemicals [1]. Drug-induced SJS cases account for 50% to greater than 80% whereas at least 80% of cases of TEN are drug-induced [1]. The cost of illness for SJS was found to be 119.49 USD per day in a study conducted in Indonesia whereas, another study in India showed 15.16 USD per day [3,4]. These differences in the treatment costs were due to different parameters they accounted like the cost of medication, diagnosis and consumables utilized during the hospital stay (see Fig. 1, Fig. 2).

Although the use of antibiotics namely cephalosporins (mainly cephalexin) has been recorded with the evolution of SJS, cefixime (a third-generation cephalosporin), has been delineated in 2 articles only [5,6]. We present a case of 40 years old male who developed SJS temporally linked to cefixime administration. In our case, the patient was a known patient who was admitted due to a road traffic accident before, this made us easier to go through his past profile and determine the offending drug timely. This case report has been reported in line with the SCARE Criteria [7].

## 2 Case description

A 40 years old male got admitted with a chief complaint of fever, cutaneous lesions with burning and itching sensation, and odynophagia.

The patient was a known patient who got admitted to the emergency ward 7 days back in our hospital after a Road Traffic accident with a GCS score of E4V5M6. He had lacerations over his head and foot with some minor bruises for which he was admitted for 3 days and was treated accordingly. During his medication, he didn't have any reactions to the treatment. During his discharge he was prescribed 10 days of antibiotics cefixime 200 mg b.d., and painkillers (combination of paracetamol and tramadol, 325 mg + 37.5 mg o.d.) only if required. During his presentation, he denied taking any painkillers and was on antibiotics solely for 6 past days after which he developed cutaneous manifestations. On, taking history, he didn't have such manifestations before, his family members didn't have any such problems, neither did he have any known autoimmune disease, allergy history, or active malignancy, nor did he have any significant drug history and for the past 6 days, he wasn't under any drug except antibiotic, cefixime.

On examination, multiple lesions were present which were hyperpigmented and reddish-purple target macules and papules over his face, trunk, back, and upper limbs with involvement of <10% BSA. The rash was erythematous with the presence of blisters and necrotic core along with the presence of the Nikolsky sign. In the oral cavity, there was an ulcerative lesion along with the hemorrhagic crusty erosion of the mucosa of lips and tip of the nose and also a whitish lesion over the dorsum of the tongue. Ocular involvement and genitalia were spared. The rash was present along with fever (103° F), odynophagia and dysphagia. The prognosis assessing SCORTEN score was 2 (mortality rate of 12.1%) at the time of admission. His vital signs were within normal limits.

On investigation, haemoglobin was 12.4 g/dl (normal male: 13–18 g/dl), ESR was 37 mm in 1st hour (male: 0–10 mm in 1st hour), random blood sugar (RBS) was 10.10 mmol/l (adult: 3.67–7.8 mmol/l). Other parameters were in the normal range.

Management of the patient was done with prednisolone tablet 20mg in tapering dose for 12 days, fexofenadine 120 mg o.d. and chlorpheniramine 4 mg for 10 days, omeprazole 20 mg b.d. for 12 days, paracetamol 500 mg t.d.s, nystatin oral drops for 15 days and gargling with 1% viodin with lukewarm water 8 hourly. Concerning therapeutic drug monitoring and periodically improvement of the patient's health condition, he was discharged after 12 days of hospital stay with the recovery of skin and some residual dysphagia. He was advised and counseled regarding his condition and precaution to be taken for future antibiotic use. Unfortunately, after the discharge of the patient, his follow-up couldn't be maintained. This was due to the lack of compliance from the patient's side, despite the advice for three follow-up routines after 2 days, one week and one month respectively.

## 3 Discussion

Steven Johnson syndrome is a life-threatening immune-mediated hypersensitive reaction caused by either medications or infections [1]. It accounts for around 30–50% of cutaneous drug reactions [8]. Some risk factors have been identified for SJS which include HIV, active malignancy, collagen diseases [1], and certain genetical predispositions like the HLA-B*1502 allele associated with carbamazepine [9] and even herpes simplex virus, bacteria like mycoplasma and measles vaccine [10].

Steven Johnson syndrome (SJS) and toxic epidermal necrolysis (TEN) are opposite ends of a spectrum of diseases arising usually from an adverse reaction to medications. It can be differentiated by [1]:

1. SJS: A minor form of Toxic Epidermal Necrolysis (TEN), with <10% BSA detachment
2. Overlapping SJS/TEN: BSA detachment 10–30%
3. TEN: BSA detachment >30%

There is a wide range of adverse drug reactions including SJS/TEN, hyper-sensitivity syndrome (HSS), anaphylaxis, and serum sickness cutaneous vasculitis [11]. Among the most common drug reactions include penicillin in antibiotics, carbamazepine in antiepileptics and allopurinol in gout treatment in the Asian community [12]. The onset of the reaction to be present after administration of the offending drug is around 4–28 days. In our case, the patient was under Cefixime for 6 days after which cutaneous manifestations were seen. There is evidence regarding the association of Cephalosporin as the culprit for SJS/TEN. This group of drugs is considered the 5th most common among antibiotics giving rise to SJS/TEN [13].

SJS is a fatal condition, with a global mortality rate stretching between 10% and 34%, thus warrants early identification and treatment based on a multidisciplinary approach [14]. The mortality rate in SJS-TEN can be predicted with SCORTEN scale, which is calculated by parameters such as the age of the patient, tachycardia, raised levels of urea, serum glucose, bicarbonates and total body surface area involved [15]. A score greater than 4 stipulates a mortality rate of 90% [15]. In a recent cohort study of 59 patients, SCORTEN scale was modified by adding two more parameters: the interval between the onset and treatment commencement at the hospital (≧8 days) and respiratory disorder within 48 h after admission, which aided in better prediction of the prognosis [14].

The first step in its management is to identify the culprit drug and stop its use. Other is symptomatic, with special attention to airway and hemodynamic stability, wound care and pain alleviation measures [15]. Among medical therapy include corticosteroids, cyclosporine, intravenous immunoglobulin (IVIG), and TNF- α inhibitors [16]. The role of steroid use in SJS/TEN management is still controversial. In a meta-analysis of immunomodulatory therapy using 96 studies, three analyses pointed out the benefit of steroid use in prognosis. It also showed a promising result using cyclosporine whereas other therapies including IVIG didn't have any beneficial role [17]. In a randomized controlled trial of 91 patients with TNF- α inhibitor (etanercept) versus steroid in SJS-TEN, the mortality rates in both the groups were below the SCORTEN-based mortality rate prediction (8.3% in the etanercept and 16.3% in the steroid groups, compared to 17.7% in the SCORTEN) [18]. Further research should be conducted for etanercept use in SJS/TEN management. A recent meta-analysis showed cyclosporine and immunoglobulins plus corticosteroids having a significant association with fewer deaths than SCORTEN prediction [19]. Another meta-analysis showed IVIG use in a higher dose to be effective (at least 2 g/kg or 3 g/kg of immunoglobulin) rather than a lower dose [20] giving a field for future study.

The strength of the article is that it has covered to-date therapy modalities preferred for SJS/TEN management along with a modified SCORTEN score for determining the prognosis of the disease. It has also added a shred of evidence to the literature of cefixime responsible for SJS, which to date only two reports have been published. On the other hand, the weakness of the article is that it has covered only a single case, a robust study with a larger sample size must be conducted in Bangladesh to make a treatment guideline for its management.

## 4 Conclusion

Cephalosporin group like cefixime is a commonly prescribed drug in developing countries due to its efficacy and cost-effectiveness. Therefore, physicians must beforehand be mindful of the consequences of its use and advice patients to visit the hospital with even the slightest cutaneous manifestation. SJS can result in fatal, life-threatening conditions if not treated timely.

## Provenance and peer review

Not commissioned, externally peer-reviewed.

## Conflicts of interest

None.

## Sources of funding

None.

## Ethical approval

N/a.

## Consent

Written informed consent was obtained from the parents of the patient for publication of this case report and accompanying images.

A copy of the written consent is available for review by the Editor-in-Chief of this journal on request.

## Author contribution

All authors contributed equally to this article.

## Research registration (for case reports detailing a new surgical technique or new equipment/technology)

N/a.

## Guarantor

First author.

## Ethical approval

Doesn't require.

## Sources of funding

Not sponsored.

## Author contribution

All authors contributed equally.

## Registration of research studies

1. Name of the registry: Not required.

2. Unique Identifying number or registration ID:

3. Hyperlink to your specific registration (must be publicly accessible and will be checked):

## Guarantor

Abhigan babu shrestha.

## Consent

Written informed consent was obtained from the patient's parents for publication of this case report and accompanying images. A copy of the written consent is available for review by the Editor-in-Chief of this journal on request.

## Declaration of competing interest

No conflicts.
